# Porous Hybrids Structure between Silver Nanoparticle and Layered Double Hydroxide for Surface-Enhanced Raman Spectroscopy

**DOI:** 10.3390/nano11020447

**Published:** 2021-02-10

**Authors:** Su-Bin Lee, Seung-Min Paek, Jae-Min Oh

**Affiliations:** 1 Department of Energy and Materials Engineering, Dongguk University-Seoul, Seoul 04620, Korea; sban0103@naver.com; 2 Department of Chemistry, Kyungpook National University, Daegu 41566, Korea

**Keywords:** silver nanoparticle, layered double hydroxide, surface-enhanced Raman spectroscopy, porous structure

## Abstract

Silver nanoparticle (AgNP), in terms of antibacterial, catalytic, electronic, and optical applications, is an attractive material. Especially, when prepared to furnish sharp edge and systematic particle orientation on the substrate, AgNPs can take advantage of surface-enhanced Raman spectroscopy (SERS). In this research, we suggested a synthetic method to immobilize the AgNP on metal oxide by utilizing Ag-thiolate and layered double hydroxide (LDH) as precursor and template, respectively. The layer-by-layer structure of LDH and Ag-thiolate transformed through reductive calcination to metal oxide and AgNP array. Physicochemical characterization, including powder X-ray diffraction, N_2_ adsorption–desorption, microscopies, and X-ray photoelectron spectroscopy, revealed that the AgNP with sufficient crystallinity and particle gap was obtained at relatively high calcination temperature, ~600 °C. UV-vis diffusion reflectance spectroscopy showed that the calcination temperature affected particle size and electronic structure of AgNP. The prepared materials were subjected to SERS tests toward 4-nitrothiophenol (4-NTP). The sample obtained at 600 °C exhibited 50 times higher substrate enhancement factor (SEF) than the one obtained at 400 °C, suggesting that the calcination temperature was a determining parameter to enhance SERS activity in current synthetic condition.

## 1. Introduction

Silver nanoparticle (AgNP) is one of the most fascinating nanomaterials both in academic and industrial fields. Due to the high antibacterial effect, AgNP is applied in food packaging [1,2], consumer products [3], fabrics [4], etc. It is also utilized in drug delivery [5], environmental application [6], and agriculture [7]. AgNP is also widely studied as a decomposing catalyst for dyes [8,9,10] as the electrons released from Ag ion are easily exchanged between electron donor and dyes [11]. In addition to the above-mentioned applications, the surface plasmon resonance (SPR) property of AgNP is known to enhance the Raman signal of certain molecules, which is often referred to as surface-enhanced Raman spectroscopy (SERS).

Since the SERS effect was first observed with pyridine which was adsorbed on rough Ag electrode [12], various techniques have been suggested to develop substrates with enhanced SERS properties. The SERS effect is known to be mediated by two principles, electromagnetic mechanism (EM) and chemical mechanism (CM) [13,14,15]. The EM is a long-range effect originated from an enhanced electromagnetic field and it depends on the morphology of the substrate. On the other hand, the CM is a short-range effect related to the charge transfer between molecule and substrate. It is generally accepted that EM dominates the SERS performance, and thus the research has mainly focused on the development of sensor materials such as AgNPs. For effective signal enhancement, several conditions are required, as follows: (i) To maximize the localization of irradiated light, AgNP should have a nanostructure, also referred to as hotspots [16,17], and (ii) for effective plasmon effect, target molecules should be located within 10 nm distance. The hotspot effect was known to be amplified at the sharp edge of AgNP, and therefore various shaped nanoparticles like nanocube, nanoprism, and nanorod have been studied [18,19,20]. For example, star-shaped AgNP with expanded arms, which was synthesized by reducing Ag^+^ under the existence of neutral hydroxylamine and citrate, showed high SERS performance [21]. Rod-shaped AgNP with controlled aspect ratio prepared by Gopchandran et al. [22] exhibited tuned longitudinal surface plasmon resonance in the range of (400–700) nm as well as effective SERS activity. In addition, the inter-particle distance is an important factor in hotspot evolution and signal enhancement. AgNP or AuNP assembled nanostructures showed signal amplification that was inversely dependent on the inter-particle distance [23]. It was suggested that silver nitrate which was systematically incorporated into block-copolymer could be reduced to silver nanoclusters with appropriate gap distance, giving rise to high sensitivity and reproducibility in SERS [24]. Recent developments of the exquisite fabrication of nanostructure utilizing self-assembly or lithography have enabled the SERS application field of AgNP or AuNP [25]. Currently, the SERS-related study is mainly carried out on the combination of hotspots by the plasmonic nanoparticles and substrate for particle array [26]. For instance, AgNP sputtered on rough cicada wing as a substrate was suggested, and the prepared AgNP-cicada wing nanomodule structure facilitated molecule detection through the SERS effect [27]. Similarly, bio-films such as chitosan were utilized to arrange the AgNP, preserving an appropriate inter-particle gap as well as particle morhpology [28]. The prepared AgNP-chitosan film exhibited high Raman signal against adenine. Jelinek et al. [29] produced AgNPs on polydimethylsiloxane film which contained amphiphilic ascorbic acid derivatives. The ascorbic acid reduced Ag^+^ to develop the AgNP and the particle arrangement occurred simultaneously. Thus, fabricated SERS film showed high Raman signal against 4-aminothiopehnol as well as a bacterial sample. Recently, a combination of covalent organic framework and AuNP was reported as another example of NP-substrate combination to achieve sensitivity in SERS recognition [30].

Recently, we have suggested a method to grow highly anisotropic Ag nanosheet between clay layers to take advantage of the sharp edges as hotspots [31,32]. As a proof-of-concept, the papers suggested that thin Ag nanosheets could be grown between the clay layers [31]. In the successive work, the potential application of the prepared AgNP-clay for SERS detection was demonstrated with a probe molecule, rhodamine 6G dye [32]. However, the previous works focused on the physicochemical characterization on AgNP-clay structure, lacking quantitative data to address how the AgNP-clay activated the SERS performance. Furthermore, in the previous work, it was not comprehended systematically how the optical property of AgNPs could be controlled by synthetic condition and what property is required to optimize the SERS performance. Therefore, in this study, we are going to focus on the optical and structural properties of the product depending on the synthetic conditions. The structural parameters are strongly related to particle size or crystallinity of AgNP, affecting the optical properties, like SERS behavior. Moreover, structural factors like inter-particle gap and orientation of AgNP on clay substrate are related to the porous structure, which influences the accessibility to the probe molecule.

**Scheme 1:**
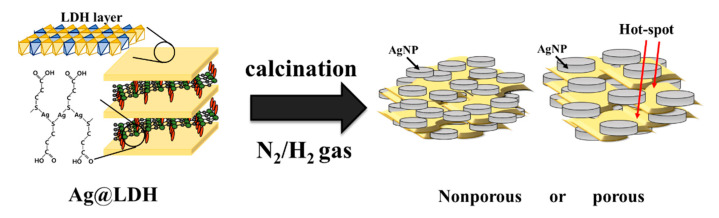
Schematic of the production of layered double oxide (LDO) and AgNP hybrid (Ag@LDO), starting from the layer-by-layer structure of layered double hydroxide (LDH) and Ag-thiolate complex with carboxylate moiety (Ag/3-MPA).

Layered double hydroxide (LDH), which is also referred to as anionic clay [33,34], was selected as a substrate to grow and orient the AgNP. LDH has a positively charged layer (general formula of M(II)_1−x_M(III)_x_(OH)_2_^x+^) with ~0.5 nm thickness, and a diameter of tens of hundreds of nanometers. The layers are stacked through sandwiching anionic molecules through electrostatic interaction. As an Ag-precursor, Ag-thiolate complex having 2-dimensional heterocatenated structure [35,36,37] and anionic functional group was utilized. As described in Scheme 1, Ag-thiolate with carboxylate moiety was intercalated into LDH in a layer-by-layer manner. Upon reductive calcination, the LDH layer loses hydroxyl groups and changes to metal oxide, i.e., layered double oxide (LDO) [38,39,40]; at the same time, Ag-thiolate transforms to AgNP with thin-layer shape due to the template effect of LDH [27]. Thermal treatment was carried out at three different temperatures (400, 500, and 600 °C), at which the organic moiety completely combusted and the structure of LDO was well-preserved. Temperature-dependent physicochemical properties such as crystallinity, particle size, porosity, and chemical environment of AgNP in the final samples were systematically investigated. Furthermore, the optical property of AgNP depending on the temperature was quantitatively analyzed with Ultraviolet-visible (UV-vis) diffuse reflectance spectroscopy and Kubelka-Munk analyses. Finally, the SERS effect was evaluated utilizing 4-nitrothiophenol (4-NTP) as a target molecule and quantitatively analyzed based on substrate enhancement (SEF) factor.

## 2. Materials and Methods

### 2.1. Materials

Magnesium nitrate hexahydrate (Mg(NO_3_)_2_∙6H_2_O), aluminum nitrate nonahydrate (Al(NO_3_)_3_∙9H_2_O), silver nitrate (AgNO_3_), and 3-mercaptopropionic acid (3-MPA) were purchased from Sigma-Aldrich Inc. (St. Louis, MO, USA). Sodium hydroxide (NaOH), tetrahydrofuran (THF), and ethyl alcohol (EtOH, 94.5%) were obtained from Daejung Chemicals & Materials Co., Ltd. (Siheung, Korea). The target molecule, 4-nitrothiophenol (4-NTP), was acquired from Tokyo Chemical Industry Co., Ltd. (Tokyo, Japan).

### 2.2. Preparation of Ag/3-MPA

To prepare Ag/3-MPA, 0.015 mol of AgNO_3_ was dissolved in mixed solvent of THF/EtOH (225 mL). Next, 7.8 mL of 3-MPA was dissolved in 150 mL of THF then dropwise added to the AgNO_3_ solution. The reaction was carried out at 80 °C under reflux condition. After 24 h of vigorous stirring, the obtained ivory-colored precipitates were centrifuged and successively washed with THF and EtOH. The product was lyophilized for further reaction.

### 2.3. Hybridization of Ag@LDH and Prepartion of Ag@LDO

To hybridize the Ag/3-MPA with LDH in a layer-by-layer manner, 0.005 mmol of Ag/3-MPA (formula weight: 212.10 g/mol) was first dispersed in the decarbonated water (150 mL) with vigorous stirring for 2 h. Then, 50 mL of NaOH solution (0.4 mol/L) was dropwise added to the Ag/3-MPA suspension in order to deprotonate the carboxyl group. The obtained exfoliated Ag/3-MPA colloid was subjected to further hybridization. Both the metal solution (0.005 mol of Mg^2+^ and 0.0025 mol of Al^3+^ in 100 mL decarbonated water) and NaOH solution (0.02 mol of NaOH in 50 mL decarbonated water) were simultaneously added to the exfoliated Ag/3-MPA colloid until ~pH 9.5 under N_2_ purging. After 24 h of stirring, the obtained Ag@LDH was centrifuged, washed with decarbonated water, and then lyophilized. The obtained Ag@LDH was thermally treated in the tube furnace at different temperatures, 400, 500, and 600 °C, respectively for 2 h. Mixed reductive gas, N_2_/H_2_ (9:1), was flowed at a 15 mL/min rate during the heat treatment. The calcined Ag@LDH was designated as Ag@LDO400, Ag@LDO500, and Ag@LDO600, according to the calcination temperature.

### 2.4. Characterization

Powder X-ray diffraction (PXRD) patterns were investigated using Ultima IV (Rigaku, Tokyo, Japan) with Cu–Kα radiation (*λ* = 1.5406 Å), with a scanning rate of 3°/min. Adequate amounts of sample powder were located on the sample holder and mounted with a flat glass slide. Crystallite size was calculated with Scherrer’s equation as described below (Equation (1)) [41]:

(1) $$\tau =\;\frac{0.9\lambda }{\beta cos\theta }$$

where *τ*: mean crystallite size in Å, *θ*: X-ray wavelength of 1.5406 Å, *β*: full-width at half maximum intensity, and *θ*: Bragg angle.

N_2_ adsorption–desorption isotherms were obtained with Belsorp-mini (BEL Japan, INC., Osaka, Japan). Samples were pre-treated at 150 °C for 24 h under vacuum condition for degassing. Specific surface area and pore size distribution were calculated based on the Brunauer–Emmett–Teller (BET) theory and the Barrett–Joyner–Halenda (BJH) method, respectively. Field-emission scanning electron microscopic (FE-SEM) images were obtained with JSM-7100F (JEOL Ltd., Tokyo, Japan) at an acceleration voltage of 15 kV. Powder samples were directly spread on sticky carbon tape and sputtered with Pt/Pd plasma for 60 s. Field-emission transmission electron microscopy (FE-TEM) and energy dispersive spectroscopy (EDS) mapping were carried out with JEM-F200 (JEOL Ltd., Tokyo, Japan) with 200 kV of accelerating voltage. Each sample was thoroughly dispersed in EtOH (0.1 mg/mL) and sonicated for 10 min. Aliquot of suspension was drop-cast on Cu-grid (200-square mesh grid with carbon film, Ted Pella, CA, USA) and dried under ambient condition. X-ray photoelectron spectroscopy (XPS) was measured using K-alpha X-ray photoelectron spectrometry (Thermo Fisher Scientific, Waltham, MA, USA).

The electronic structure was analyzed by UV-vis diffusion reflectance spectroscopy (EVOLUTION 220, ThermoFisher Scientific, Waltham, MA, USA) equipped with an integrating sphere (ISA-220) in the wavelength from 190 to 1100 nm, with integration time of 0.86 s, 1.00 nm of data interval, and 69.8 nm/min of scan speed. First, approximately 0.1 g of powder samples were located in a quartz holder without any treatment. To eliminate the background interference, standard BaSO_4_ was utilized. To determine the band gap energy of Ag@LDOs, we converted the absorbance to Kubelka-Munk reflectance value using Tauc formula (Equation (2)) [42]:

(2) $${\left(\alpha h\nu \right)}^{1/\gamma }\;=B\left(h\nu -E_{g}\right)$$

where *h*: the Planck constant, *α*: energy-dependent absorption coefficient, *ν*: the photon’s energy, *E_g_*: band-gap energy, and *B*: constant. The *γ* factor is the nature of the electron transition and equals to 1/2 or 2 for the direct and indirect transition band gaps, respectively. The *E_g_* value was determined by the intercept of a straight line that rises steeply in the low-energy region in the hν(eν) vs. [F(R∞)hν]^2^ plot.

### 2.5. Surface-Enhanced Raman Scattering Spectroscopy (SERS)

To investigate the SERS effect of the sample, Raman spectroscopy was carried out with 4-NTP. First, silicon wafers were cut into small pieces (of approximately 1 × 1 cm size). Four pieces of silicon wafers were cleaned with piranha solution (H_2_SO_4_:H_2_O_2_ = 3:1 volume ratio). Then, 0.01 g of each Ag@LDO was dispersed in 10 mL of deionized water and 50 μL of each suspension was dropped on the three respective silicon wafers. The sample was dried on a hot-plate (~50 °C). Bare wafer was utilized as a reference sample. Target molecule, 4-NTP, was prepared in solutions with concentration (10^−4^–10^−7^) mol/L and 20 μL of 4-NTP solution was dropped on silicon wafers with or without Ag@LDO. After drying solvent under ambient condition, Raman spectra were recorded at 514 nm using LabRam Aramis (Horiba Jobin Yvon, Kyoto, Japan). An Ar-ion laser with ~10 uW of power and 5 s of acquisition time was used to measure the excitation spectra in the range (200–3000) cm^−1^. To analyze the performance of the SERS substrate, we calculated the substrate-enhancement factor (SEF) of Ag@LDO, using Equation (3):

(3) $$SEF=\;\frac{I_{SERS}}{I_{RS}}\;\times \;\frac{C_{RS}}{C_{SERS}}\frac{V_{RS}}{V_{SERS}}$$

Here, *I_SERS_* and *I_RS_* indicate Raman signal intensity with and without SERS substrate, respectively. *C_RS_* is an analyte solution concentration under non-SERS condition while *C_SERS_* is an analyte solution concentration under SERS condition. *V_RS_* and *V_SERS_* means the probed volume for non-SERS and SERS conditions [43,44].

## 3. Results and Discussion

### 3.1. Crystal Strucutre of Ag@LDO

**Figure 1:**
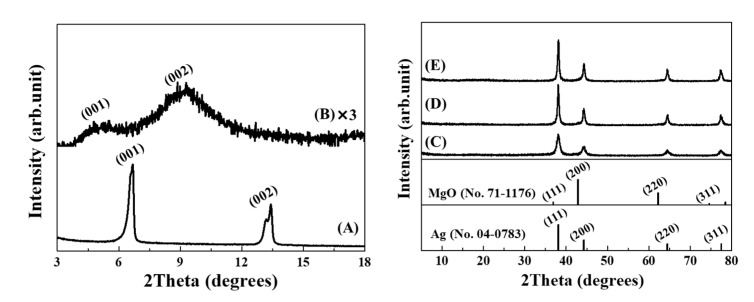
Powder X-ray diffraction (PXRD) patterns of (**A**) Ag/3-MPA, (**B**) Ag@LDH, (**C**) Ag@LDO400, (**D**) Ag@LDO500, and (**E**) Ag@LDO600.

We first investigated the structure of all the samples with powder X-ray diffraction (PXRD). It is one of the generally utilized techniques to identify the crystalline phase and to evaluate the crystallinity of materials. X-ray beam (0.5–2.5 Å) irradiated to the powder sample is diffracted at certain lattice planes which are periodically aligned. If the angle of incident X-ray satisfies a specific condition, the diffracted beams influence each other in a constructive interference manner, according to Bragg’s law, thus the inter-plane distance, i.e., d-spacing, can be calculated. In this way, crystal structure and crystallinity of powder samples can be evaluated [41,45,46]. As shown in the left panel of Figure 1, Ag/3-MPA and Ag@LDH showed characteristic diffraction patterns at the low-angle region. The diffractogram of Ag/3-MPA showed well-developed (00*l*) patterns with d-spacing 13.2 Å (Figure 1A). According to our previous research, Ag-thiolate complex with 3-MPA moiety formed quasi-hexagonal 2-dimensional layers that were interconnected through dimerization between carboxylic acids from adjacent layers [32].

Considering the thickness of the Ag-S slab of (13–14) Å, chain length of 3-MPA (6.2 Å), and hydrogen bonding between carboxylic acids (~1 Å), the d-spacing value 13.2 Å suggested successful formation of the layered Ag/3-MPA structure. After hybridization with LDH, sharp (00*l*) peaks for Ag/3-MPA disappeared and two diffraction peaks at 2θ = (5.05 and 9.14°) appeared with relative low intensity. As the d-spacing of the second one (9.1 Å) was half of the first one (18.2 Å), the two peaks were considered reciprocal signals of (00*l*) in Ag@LDH. As the single layer of LDH and Ag/3-MPA has respective thickness of 4.8 and 13.2 Å respectively, the d-spacing of the first peak could be considered as the basal spacing of Ag@LDH. Two points in the diffractogram Ag@LDH should be noted: (i) the overall intensity of diffraction in Ag@LDH was significantly reduced, compared with Ag/3-MPA, and (ii) the (002) peak was larger in intensity than the (001) peak. The first phenomena could be addressed by the different chemical interaction between layers in the Ag/3-MPA and Ag@LDH hybrid. In Ag/3-MPA, layers were strongly attached to each other through periodic hydrogen bond; on the other hand, stacking in Ag@LDH was maintained through relatively weak electrostatic interaction, resulting in relatively low ordered stacking. The second point was attributed to the super-lattice structure [31,47], and both layers from Ag/3-MPA and LDH contained periodic metal arrays in their centers and thus the strong diffraction occurred between the two layers.

After calcination, the resulting products exhibited characteristic peaks of face-centered-cubic structure silver, such as 2θ = (38.1, 44.2, 64, and 77.3°), corresponding to (111), (200), (220), and (311), respectively (JCPDS No. 04-0783). It is generally known that the thermal treatment on MgAl-LDHs at temperature range (300–600) °C results in the phase transformation to MgAl-LDO through sequential dehydration, dehydroxylation, and gasification of interlayer anion, resulting in MgO phase (JCPDS No. 71-1176) [33,48,49]. However, we did not observe any crystalline diffraction of MgO. The result implied that during calcination, the reduction of interlayer Ag/3-MPA was more dominant than the crystal growth of MgO. The peaks corresponding to Ag were broad and exhibited low intensity in Ag@LDO400. Increase in calcination temperature gradually enhanced the crystallinity and narrowed the peak width. Quantitative analysis with Scherrer’s equation (Equation (1)) revealed that the crystallite size along the (111) direction was 8.01, 15.6, and 16.7 nm, for Ag@LDO400, Ag@LDO500, and Ag@LDO600, respectively. Through these results, we determined that the calcination temperature of around 500 °C was enough to obtain crystalline AgNP in LDH.

### 3.2. Pore Structure of Ag@LDO

**Figure 2:**
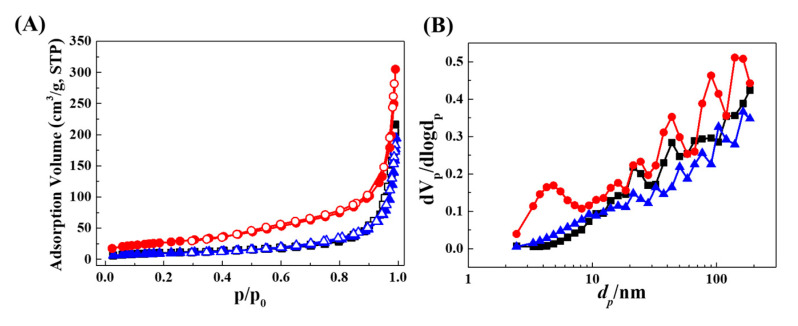
(**A**) N_2_ adsorption–desorption isotherms and (**B**) corresponding Barrett-Joyner-Halenda (BJH) pore size distribution for Ag@LDO400 (black), Ag@LDO500 (blue), and Ag@LDO600 (red). Solid and open symbols in isotherms represented adsorption and desorption branch, respectively.

Pore structure and specific surface area of Ag@LDOs were analyzed based on the N_2_ adsorption–desorption isotherms (Figure 2A). The result showed type III adsorption patterns for the low-temperature samples (Ag@LDO400 and Ag@LDO500), and type II pattern for Ag@LDO600. According to the International Union of Pure and Applied Chemistry (IUPAC) classification [50,51], type III was observed in nonporous material with weak adsorbent–adsorbate interaction, whereas type II was attributed to porous material with macropores as a majority. The results indicated that calcination at 400 or 500 °C was not effective to produce porous structure, although the crystallite size of AgNP was fairly high at 500 °C (Figure 1). This was confirmed by the pore size distribution displayed in Figure 2B. The majority of porosity in Ag@LDO400 and Ag@LDO500 was attributed to pores larger than 10 nm; on the other hand, Ag@LDO600 clearly showed mesopore contribution in the range of (2–10) nm (red symbol and line in Figure 2B). Accordingly, the specific surface area value of Ag@LDO600 (96.341 m^2^/g) was dramatically higher than those of Ag@LDO400 and Ag@LDO500 (38.08 and 35.17 m^2^/g), respectively.

The evolution of mesopores at high temperature could be related to the reduction process of Ag-thiolate. According to our previous study, reductive calcination changed layered Ag-thiolate complex to zero valency Ag with various morphology depending on the substrate [52,53]. Free Ag-thiolate powder and Si wafer-coated Ag-thiolate resulted in fibrous silver and pebble-shaped silver particles, respectively. Both results suggested that the thermal decomposition of organic moiety facilitated the Ag–Ag assembly to grow crystal and the particle arrangement was affected by the type of substrate. As Ag atoms between LDH layers were aligned in 2-dimensional direction, the crystal growth would predominantly occur along the *ab*-plane direction. When the Ag-thiolate moiety in the interlayer space of LDH is calcined under reductive air, small Ag(0) nuclei would develop upon the decomposition of organic ligand, and the adjacent Ag(0) nuclei would collectively merge together resulting in AgNP. The process is partially governed by the crystal growth, of which rate was temperature-dependent [54]. Therefore, upon increasing temperature, the neighboring Ag moiety could be gathered in a facilitated manner, resulting in the sufficient inter-particle space among AgNPs (Scheme 1). The evolution of nanopores is important in SERS application: (i) enhanced surface plasmon resonance is possible in the inter-particle space of AgNP, i.e., hotspot [55,56], (ii) nano-sized pore is advantageous for target molecules to access AgNP for attachment, and (iii) high specific surface area (~100 m^2^/g) enables the effective adsorption of target molecules at low concentrations.

### 3.3. Microscopic Structures of Ag@LDO

**Figure 3:**
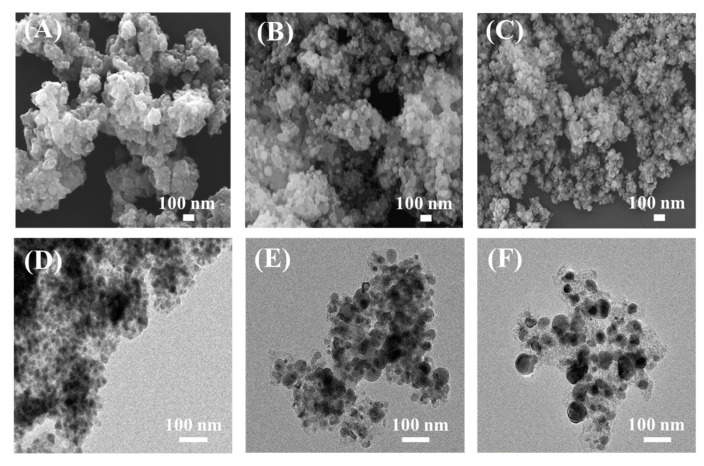
Field-emission scanning electron microscopy (FE-SEM) and field-emission transmission electron microscopy (FE-TEM) images for (**A**,**D**) Ag@LDO400, (**B**,**E**) Ag@LDO500, and (**C**,**F**) Ag@LDO600 (Upper row: SEM images, lower row: TEM images).

The size and shape of Ag@LDOs were examined by FE-SEM and FE-TEM. Figure 3A–C show that the overall morphology of samples was fairly irregular. However, upon increasing the calcination temperature, the SEM images began to show spherical particles, in particular, exhibiting small grains in Figure 3C.

The TEM images (Figure 3D–F) clearly displayed the evolution of spherical particles that were considered as AgNP. Although the Ag@LDO seemed to have large aggregates in the microscopic images, it had high colloidal stability, with less possibility to form aggregates. As shown in Supplementary Figure S1A, the aqueous suspension of Ag@LDO preserved stable colloidal state, while conventionally available Ag particles (200 mesh, Kojima Chemicals Co., LTD, Saitama, Japan) readily precipitated after dispersing in water. The hydrodynamic radius of Ag@LDO was 680.9 nm, with polydispersity index (PDI = (standard deviation of radius)^2^/(average radius)) of 0.304 (Supplementary Figure S1B). It strongly suggested that the Ag@LDO is highly dispersible in water and therefore the material can be easily drop-cast on solid substrate for SERS application. The large chunks in microscopic images were accidently formed during sample preparation. All the three TEM images showed dark particles covered by a relatively gray region. As the LDO was composed of MgO and Al_2_O_3_, oxide of light elements, it was represented as a gray-colored region. On the other hand, the strong dark contrast was ascribed to the Ag moiety.

**Figure 4:**
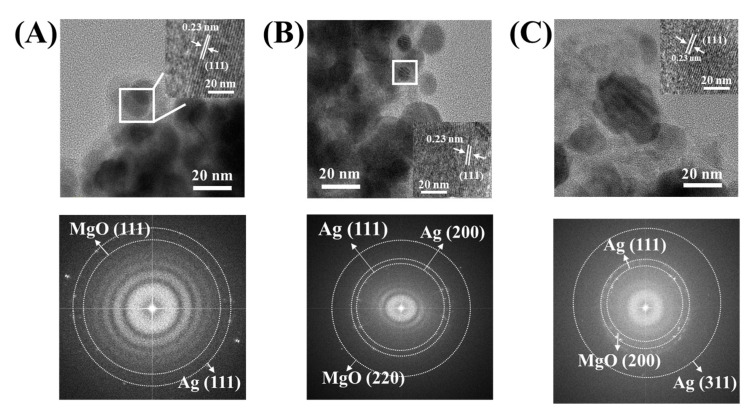
Magnified FE-TEM images and corresponding fast Fourier transform (FFT) patterns for (**A**) Ag@LDO400, (**B**) Ag@LDO500, and (**C**) Ag@LDO600.

Magnified FE-TEM images represented a clear lattice fringe in the dark particle region (Figure 4). The d-spacing of the lattice fringe was measured to be 0.23 nm, which corresponded to the (111) plane of face-centered-cubic Ag. The fast Fourier transform (FFT) images in Figure 4 showed more information on the existence of Ag or LDO. In addition to the 0.23 nm FFT (Ag (111)), spot patterns could be observed with 0.20 and 0.11 nm, which were attributed to (200) and (311) respectively, of Ag. Furthermore, the spot patterns with 0.27, 0.24, and 0.14 nm, which can be assigned to (111), (200), and (220) of MgO, suggested the co-localization of LDO with Ag nanoparticles. The high-angle annular dark-field (HAADF) images and energy dispersive spectroscopy (EDS) mapping corroborated the co-localization of LDO (MgO + Al_2_O_3_) and AgNP (Supplementary Figure S2).

Upon increasing the calcination temperature, the estimated particle size of Ag increased significantly. The average particle size and standard deviation calculated from TEM images were 9.94 ± 1.69, 19.81 ± 4.79, and 25.13 ± 3.59 nm, for the Ag@LDO400, Ag@LDO500, and Ag@LDO600, respectively. Different from the crystallite size analyses, the Ag@LDO600 had larger average size of AgNP than did Ag@LDO500. This might be attributed to the different particle arrangement at 500 and 600 °C, although the crystal growth in the two conditions was comparable. The crystal merging was expected at 600 °C, which would result in the development of inter-particle pores. This was also supported by the inter-particle distance in the Ag@LDO600 sample and the result was parallel to the pore size distribution (Figure 2B).

### 3.4. Chemical Environments of Ag@LDO

**Figure 5:**
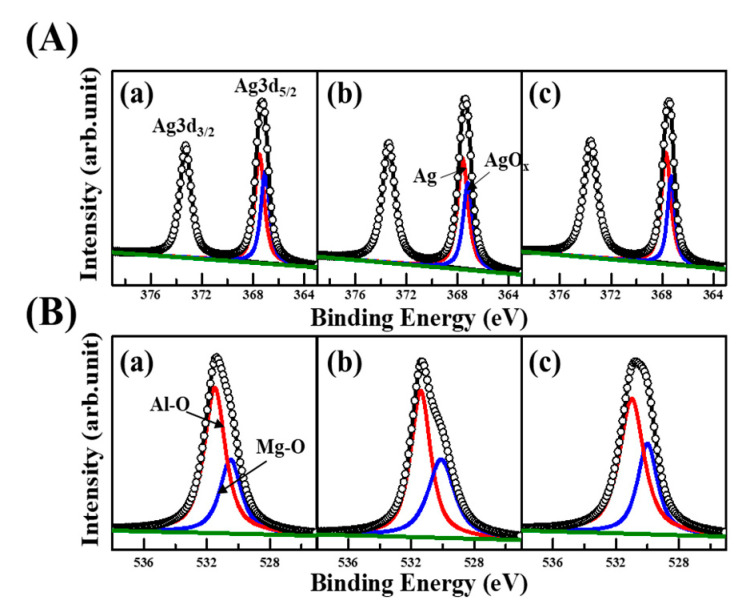
X-ray photoelectron spectroscopy (XPS) of (**A**) Ag 3d and (**B**) O 1s electrons for (**a**) Ag@LDO400, (**b**) Ag@LDO500, and (**c**) Ag@LDO600. Black line: observed spectrum, open circles: summation of separated peak, red line: Ag(0) or Al-bound O, blue line: oxidized Ag or Mg-bound O, green line: background for peak separation.

The XPS spectra focusing on Ag 3d and O 1s electrons were analyzed to examine the chemical environment in Ag@LDO samples. Figure 5A shows the XPS signals of Ag 3d doublet (3d_3/2_ and 3d_5/2_). The Ag 3d_3/2_ and Ag 3d_5/2_ peaks appear at around 373 and 367 eV respectively, which are comparable with the previously reported binding energy of AgNP [57,58,59]. According to the National Institute of Standards and Technology (NIST) database for XPS [60], the binding energy of Ag 3d_5/2_ is dependent on the oxidation state. The Ag 3d_5/2_ electrons in zero-valency Ag, monovalent Ag_2_O, and divalent AgO were reported to have binding energies in the range (367.9–368.3), (367.7–368.4), and (367.3–368.1) eV, respectively.

The tendency exhibited increasing binding energy for decreasing oxidation state. Based on this database, we could assign the separated peaks to zero valency Ag (red Line) and oxidized Ag (blue line). The partial oxidation of AgNP at particle surface was often reported in the literature. For example, the Ag composited with reduced graphene had shifted Ag 3d binding energy due to the slight oxidation of AgNP [57]. The AgNP with silica coating was also reported to have both metallic and oxide form of Ag [61]. Another research from Makita et al. [62] suggested that the surface oxidation on AgNP gradually proceeded during the period of exposure in air. We also observed that the Ag nanosheets grown in the nano-clay layer contained partial positive charge, due to the surface oxidation [32].

Quantitative calculation on the binding energy of Ag 3d_5/2_ exhibited an increasing pattern according to calcination temperature: 367.48, 367.58, and 367.68 eV of Ag (red lines in Figure 5A), and 367.08, 367.15, and 367.25 eV for oxidized-Ag (blue lines in Figure 5A) for Ag@LDO400, Ag@LDO500, and Ag@LDO600, respectively. Higher binding energy in Ag@LDO600 than the others was the result of efficient reduction in Ag, and the low portion of surface. Both PXRD and TEM analyses confirmed that the crystallite size and particle size of AgNP increased with the calcination temperature. Larger particles tended to lower the portion of oxidation at the surface, resulting in higher binding energy.

In addition to the Ag analysis, we also examined the binding energy of O 1s. The peaks could be separated into two peaks, of which the binding energy was found at 531.1 and 530.0 eV, respectively (Figure 5B). The higher binding energy was attributed to the Al-bound oxygen, and the low-energy peak was assigned to Mg-bound oxygen. The result suggests that MgO and Al_2_O_3_ co-exist in Ag@LDOs in amorphous state.

### 3.5. Electronic Structure of Ag@LDO

**Figure 6:**
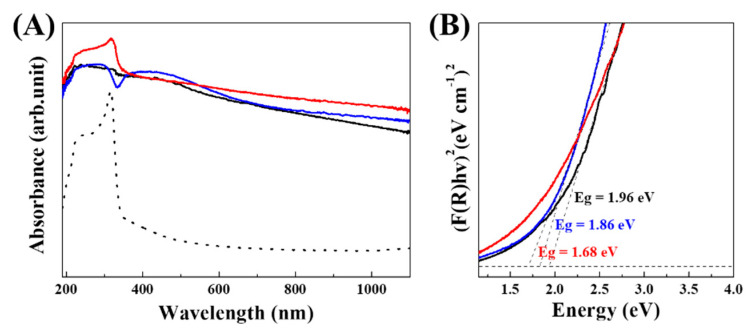
(**A**) UV-vis diffuse reflectance spectra of Ag powder (dot line) and Ag@LDOs and (**B**) Kubelka-Munk-transformed reflectance spectra of Ag@LDOs (black line: Ag@LDO400, blue line: Ag@LDO500, and red line: Ag@LDO600). No offset was set among graphs.

To evaluate the optical property and electronic structure, UV-vis diffuse reflectance spectra of the Ag@LDOs were obtained utilizing bulk silver powder as a reference. All the spectra showed intense absorption in the wavelength range (200–350) nm (Figure 6A), which was attributed to the SPR of Ag, as reported previously [63]. The spectra of Ag@LDOs characteristically showed noticeable absorption pattern over the entire visible light range, while the spectrum of bulk Ag powder did not show discernable absorption at visible light. It may be attributed to the different optical property between bulk Ag and AgNPs. Bulk Ag mostly reflect visible light; on the other hand, AgNPs with appropriate distribution in LDO matrix could have a LSPR effect [64]. The absorption intensity at the visible light region is related to the particle size. According to the previous report, particle size decrease of AgNP increased the damping parameter, giving rise to reduced absorption intensity [65]. The highest absorption of Ag@LDO600 may be strongly related to its largest particle size of AgNP, as observed in TEM (Figure 3D–F).

The band gap of AgNPs in LDO matrix was calculated by Kubelka-Munk conversion. As shown in Figure 6B, the band gap clearly decreased with increasing calcination temperature, 1.96, 1.86, and 1.68 eV for Ag@LDO400, Ag@LDO500, and Ag@LDO600 respectively (Figure 6B), suggesting the reverse-proportional relationship between band gap and particle size. This tendency was well-addressed by the quantum size effect [66,67]. Similar size band gap relationship was reported by Gharibshahi et al. [68]. They prepared AgNP in polyvinyl pyrrolidone (PVP) matrix by thermal treatment and showed the reverse relationship between particle size (7.88, 5.57, and 4.61 nm) and band gap (2.75, 2.81, and 2.83 eV), respectively. The UV-vis spectroscopy confirmed that the Ag@LDO contained AgNPs with SPR effect and suggested that the calcination condition would influence the optical property of the developed AgNP.

### 3.6. SERS Effect of Ag@LDO

**Figure 7:**
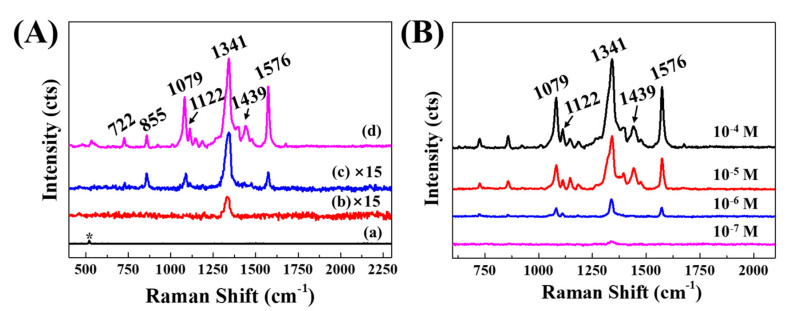
Raman spectra of (**A**) 4-NTP (10^−4^ mol/L) on (**a**) bare Si wafer, (**b**) Ag@LDO400, (**c**) Ag@LDO500, and (**d**) Ag@LDO600, and (**B**) 4-NTP with various concentrations on Ag@LDO600. All spectra were taken using a 514 nm Ar-ion laser, ~10 μW power, single 5 s accumulation. *: Raman signal for silicon substrate.

To evaluate the potential of Ag@LDOs as SERS material, and to examine the effect of calcination temperature on the efficiency, we measured the Raman spectroscopy of 4-NTP with or without Ag@LDOs. First, we focused on the qualitative SERS effect depending on the calcination temperature in Ag@LDO. Raman signal of 4-NTP was characterized by the distinctive peak at 1336 cm^−1^, attributed to the symmetric stretching of the NO_2_ group [69]. Figure 7A(a) shows that the 4-NTP solution (10^−4^ mol/L) dropped on bare silicon wafer did not show any Raman signal for the molecule. On the other hand, Raman signals of 4-NTP (10^−4^ mol/L) could be clearly observed on Ag@LDO, possibly due to the SERS effect which was dependent on the calcination temperature. The Ag@LDO600 exhibited the highest peak intensity, compared with the others. This might be due to the high crystallinity of AgNP, large specific surface area, and the hotspots developed by nano-sized pores in Ag@LDO600. As discussed before, increasing calcination temperature caused the Ag@LDOs to develop highly crystalline Ag with sufficient inter-particle distance to have considerable specific surface area. We would like to highlight again that the immobilization of AgNP in LDO substrate with appropriate spacing (Scheme 1) was the key point to producing hotspots and mesopores, giving rise to the efficient SERS phenomenon in Ag@LDO600.

To find the detection limit of Ag@LDO600, various concentrations of 4-NTP, from 10^−4^ to 10^−7^ mol/L, were applied on the substrate. Figure 7B shows that the characteristic Raman peaks of 4-NTP were revealed at concentrations as low as 10^−6^ mol/L. Typically, three distinctive peaks at 1576, 1341, and 1079 cm^−1^ were observed, which were attributed to C–C stretching vibration, NO_2_ symmetric vibration, and C–S stretching vibration respectively [70,71], at 10^−6^ mol/L. The peak corresponding to C–C stretching vibration (1439 cm^−1^) was observed until 10^−5^ mol/L. Furthermore, peaks at 1112, 855, and 722 cm^−1^ (C–H bending, C–H wagging, and C–S wagging vibrations, respectively) were observed at 10^−6^ mol/L concentration. From these results, we could suggest that the AgNPs were well-developed by calcimining Ag@LDH at 600 °C to have physicochemical properties for the SERS effect.

The quantitative analysis on SERS effect depending on calcination temperature was carried out by calculating SEF (Table 1). The SEF values toward νCC (C–C stretching) Raman signal clearly enhanced with increasing calcination temperature, showing 1.5 × 10^3^, 2.1 × 10^4^, and 8.3 × 10^4^ for Ag@LDO400, Ag@LDO500, and Ag@LDO600, respectively.

The results confirmed that the higher calcination temperature was advantageous in SERS performance. It also suggested that well-developed AgNP and mesopores by LDO substrate played important roles in Raman signal enhancement. The SEF value of Ag@LDO600, 8.3 × 10^4^, was comparable with the previously reported SEF of nanostructures toward 4-NTP. Hong and Li synthesized the AuNP in different sizes and examined the SERS effect toward 4-NTP according to particle diameter. The SEF value increased with particle size, and the SEF value was maximized at 1.3 × 10^4^ [72]. Au nanotriangles in the presence of phospholipid vesicles showed effective SERS performance toward 4-NTP (SEF = 2.76 × 10^4^) [73]. Barman et al. [74] exhibited that the SEF of Au nano-stars prepared by the seed-mediated method was 3.4 × 10^4^ toward 4-NTP. On the other hand, binary nanostructure consisting of Ag and Pd was reported to have effective SERS performance with SEF = 2.1 × 10^5^ for 4-NTP [75]. The literature comparison confirmed that the Ag@LDO obtained at 600 °C of calcination had well-developed AgNP, appropriate distribution in LDO matrix, and mesopores, resulting in high SERS performance.

## 4. Conclusions

In this paper, we demonstrated the AgNP synthesis on an LDO layer. First, Ag-thiolate with carboxylate moiety was successfully intercalated between layers of LDH. Second, reductive calcination from 400 to 600 °C was performed on Ag@LDH to accompany simultaneous processes such as dehydroxylation of LDH, crystal growth of Ag, and immobilization of AgNP on LDO substrate. The crystallite size, specific surface area, pore diameter, and the average AgNPs particle size all increased according to calcination temperature, and the optimum structure was obtained at 600 °C. The UV-vis spectra revealed that all the Ag@LDOs had SPR effect; furthermore, the higher calcination temperature resulted in narrow band gap and enhanced plasmonic effect in the visible light region. Owing to the SPR effect of AgNPs and the evolution of nanopores, Ag@LDO600 showed sensitive detection of 4-NTP through the SERS effect compared with Ag@LDOs obtained at lower calcination temperatures. The SEF value of Ag@LDO increased according to the calcination temperature. Typically, Ag@LDO600 had 50 times higher SEF value than Ag@LDO400. Therefore, we conclude that a method to produce SERS-sensitive materials could be the calcination of Ag@LDH, and to produce optimum SERS performance, an appropriate temperature should be applied.

## Figures and Tables

**Table 1:** Evaluation of substrate enhancement factors in Ag@LDO. Raman signal intensity corresponding to the νCC (C–C stretching) of 4-NTP was selected for calculation. The concentration of 4-NTP at non-SERS and SERS conditions was 5.0 × 10^−2^ and 1.0 × 10^−4^ M, respectively.

	Ag@LDO400	Ag@LDO500	Ag@LDO600
I_SERS_/counts	77	1126	4338
I_RS_·C_RS_^−1^/counts·M^−1^	524.81	524.81	524.81
SEF	1.5 × 10^3^	2.1 × 10^4^	8.3 × 10^4^

## Data Availability

Data is contained within the article or supplementary material.

## Supplementary Materials

The following are available online at https://www.mdpi.com/2079-4991/11/2/447/s1, Figure S1: (A) Aqueous suspension of conventional Ag particles (200 mesh, Kojima Chemicals Co., LTD, Saitama, Japan) and Ag@LDO. The Ag particles readily precipitated within 15 min, while Ag@LDO preserved high dispersibility. (B) Hydrodynamic radius of Ag@LDO measured by dynamic light scattering (DLS) (ELSZ-1000, Otsuka, Kyoto, Japan). The hydrodynamic radius of Ag@LDO under aqueous suspension was 680.9 nm with high mono-dispersity. The polydispersity index (PDI = (standard deviation)^2^/(average radius)) was 0.304. Figure S2: High-angle annular dark field (HAADF) and energy dispersive spectroscopy (EDS) mapping images from FE-TEM on (A) Ag@LDO400, (B) Ag@LDO500, and (C) Ag@LDO600.
