# Supplementary material for: Porous Hybrids Structure between Silver Nanoparticle and Layered Double Hydroxide for Surface-Enhanced Raman Spectroscopy
Source: Nanomaterials (Basel). 2021 Feb 10;11(2):447. doi: 10.3390/nano11020447 (PMC7916476; doi:10.3390/nano11020447)
Supplement: Supplementary file 1 [file nanomaterials-11-00447-s001.pdf]

nanomaterials

Article

Porous Hybrids Structure between Silver Nanoparticle and Layered Double Hydroxide for Surface-Enhanced Raman Spectroscopy

Su-Bin Lee [1], Seung-Min Paek [2,*] and Jae-Min Oh [1,*]

[1] Department of Energy and Materials Engineering, Dongguk University-Seoul, Seoul 04620, Korea; sban0103@naver.com
[2] Department of Chemistry, Kyungpook National University, Daegu 41566, Korea
* Correspondence: smpaek@knu.ac.kr (S.-M.P.); jaemin.oh@dongguk.edu (J.-M.O.); Tel.: +82-53-950-5335 (S.-M.P.); +82-2-2260-4977 (J.-M.O.)

Citation: Lee, S.-B.; Paek, S.-M.; Oh, J.-M. Porous Hybrids Structure between Silver Nanoparticle and Layered Double Hydroxide for Surface-Enhanced Raman Spectroscopy. *Nanomaterials* **2021**, *11*, x. https://doi.org/10.3390/xxxxx

Academic Editor: Giuseppe Lazzara
Received: 31 December 2020
Accepted: 7 February 2021
Published: date

Publisher's Note: MDPI stays neutral with regard to jurisdictional claims in published maps and institutional affiliations.

Copyright: © 2021 by the authors. Submitted for possible open access publication under the terms and conditions of the Creative Commons Attribution (CC BY) license (http://creativecommons.org/licenses/by/4.0/).

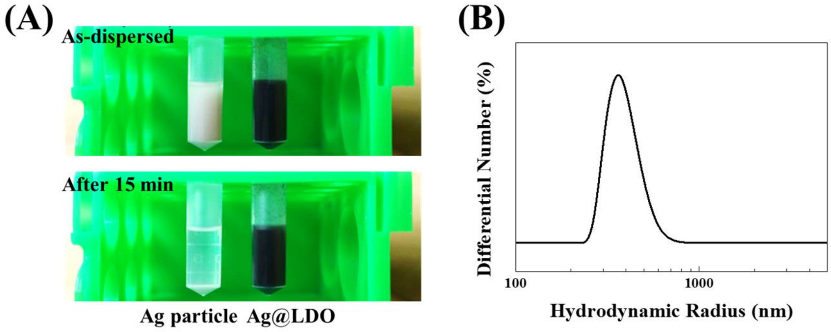

Figure S1. (**A**) Aqueous suspension of conventional Ag particles (200 mesh, Kojima Chemicals Co., LTD, Saitama, Japan) and Ag@LDO. The Ag particles readily precipitated within 15 min, while Ag@LDO preserved high dispersibility. (**B**) Hydrodynamic radius of Ag@LDO measured by dynamic light scattering (DLS) (ELSZ-1000, Otsuka, Kyoto, Japan). The hydrodynamic radius of Ag@LDO under aqueous suspension was 680.9 nm with high mono-dispersity. The polydispersity index (PDI = (standard deviation)^2/(average radius)) was 0.304.

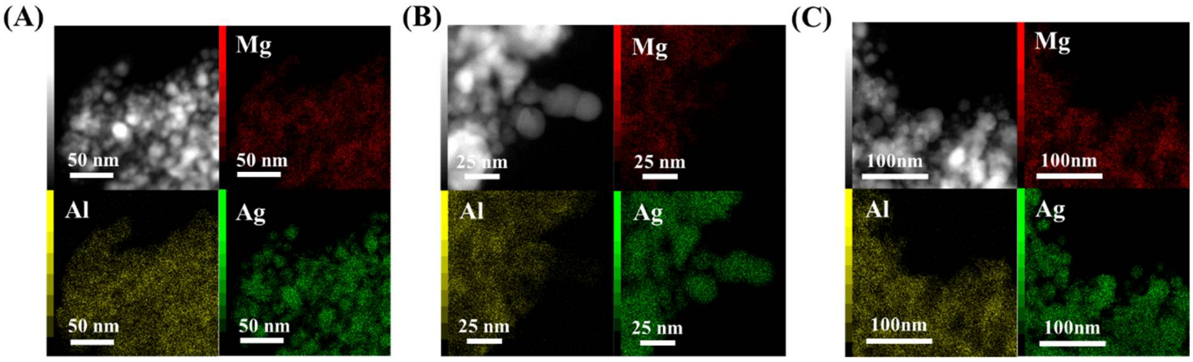

Figure S2. High-angle annular dark field (HAADF) and energy dispersive spectroscopy (EDS) mapping images from FE-TEM on (**A**) Ag@LDO400, (**B**) Ag@LDO500, and (**C**) Ag@LDO600.
